# Clinical Evaluation of Acute Exacerbation of Interstitial Lung Disease in a Single Tertiary Center: Perspectives before and after the Coronavirus Disease 2019 Pandemic

**DOI:** 10.3390/jcm13195733

**Published:** 2024-09-26

**Authors:** Ryo Takagi, Takeshi Saraya, Sho Yamada, Kei Nakajima, Kazuyuki Doi, Takatora Akizawa, Narishige Ishikawa, Nozomi Kurokawa, Fumi Kobayashi, Hiroki Nunokawa, Jumpei Aso, Yasuo Nakamoto, Manabu Ishida, Mitsuru Sada, Kojiro Honda, Keitaro Nakamoto, Saori Takata, Haruyuki Ishii

**Affiliations:** Department of Respiratory Medicine, Faculty of Medicine, Kyorin University, 6-20-2 Shinkawa, Tokyo 181-8611, Japan; ryo-m@ks.kyorin-u.ac.jp (R.T.); sho616@outlook.jp (S.Y.); kay01431@gmail.com (K.N.); doikazu0428@gmail.com (K.D.); t_akisan@ks.kyorin-u.ac.jp (T.A.); n-ishikawa@ks.kyorin-u.ac.jp (N.I.); conocococco@yahoo.co.jp (N.K.); hrk910@ks.kyorin-u.ac.jp (H.N.); j_aso@ks.kyorin-u.ac.jp (J.A.); yasuo-nakamoto@ks.kyorin-u.ac.jp (Y.N.); matsu.manabu@gmail.com (M.I.); rainbow_orchestra716@yahoo.co.jp (M.S.); h-kojiro@beach.ocn.ne.jp (K.H.); keichon2000@yahoo.co.jp (K.N.); s-takata@ks.kyorin-u.ac.jp (S.T.); h141@ks.kyorin-u.ac.jp (H.I.)

**Keywords:** acute exacerbation of interstitial pneumonia, infection, prognostic factor, seasonal predilection

## Abstract

**Background/Objectives:** Acute exacerbation (AE) of interstitial lung disease (ILD) is a major challenge. This study aimed to retrospectively investigate occurrences of AEs in patients with ILDs, including idiopathic pulmonary fibrosis (IPF), non-IPF (iNSIP: idiopathic nonspecific interstitial pneumonia), and connective tissue disease (CTD)-associated ILDs (CTD-ILDs), at a single tertiary center before and after the coronavirus disease 2019 (COVID-19) pandemic. The study aimed to clarify the seasonal and regional trends of AEs of ILDs, assess the roles of viral and bacterial infections, and identify key prognostic factors for patient outcomes. **Methods:** We conducted a retrospective review of hospitalized adult patients with AEs of ILDs from January 2019 to February 2024. **Results:** A total of 93 patients were enrolled: IPF (*n* = 42), iNSIP (*n* = 37), and CTD-ILDs (*n* = 14). The median age was 80 years (interquartile range: 74.0–86.0 years), with males comprising 64.5% (*n* = 60). AEs of ILDs predominantly occurred in winter and were particularly notable after summer 2023, coinciding with the lifting of COVID-19-related travel restrictions in Japan. Patient referrals from different areas (Northern Tama, East and/or Southern Tama, and other Tokyo metropolitan areas) were evenly distributed throughout the study period. Viral infections were detected in only two patients (SARS-CoV-2), and bacterial infections included methicillin-resistant *Staphylococcus aureus* and *Pseudomonas aeruginosa*. The Cox regression analysis identified serum lactate dehydrogenase levels ≥350 IU/L and tachypnea (respiratory rate ≥ 30 breaths per min) on admission as prognostic factors for mortality, with a hazard ratio [HR] of 2.783 (95% confidence interval [CI]: 1.480–5.235, *p* = 0.001) and an HR of 3.332 (95% CI: 1.710–6.492, *p* < 0.001), respectively. **Conclusions:** AEs of ILDs predominantly occur in winter, and viral and bacterial infections are infrequently detected. Elevated serum LDH levels and tachypnea are crucial prognostic markers for mortality. This study highlights the seasonal trend in the AE of ILD and emphasizes the importance of specific prognostic indicators in clinical practice.

## 1. Introduction

Interstitial lung disease (ILD) refers to a group of pulmonary disorders characterized by inflammation and/or fibrosis of the lung parenchyma, leading to progressive dyspnea that often culminates in end-stage respiratory failure. ILDs are subcategorized based on etiology and include connective tissue disease-associated ILD (CTD-ILD), hypersensitivity pneumonitis, drug-induced ILD, postinfectious ILD, and idiopathic interstitial pneumonias. Regarding pathophysiology, idiopathic pulmonary fibrosis (IPF) progresses with an abnormal wound healing response in genetically susceptible individuals following repeated alveolar epithelial injury, and the biological pathways of CTD-ILD are poorly understood.

In United States, ILDs, including IPF, affect approximately 0.21% of the population. The prevalence of IPF specifically is estimated at around 14.0 to 27.9 per 100,000 people. The burden of ILDs, including progressive forms, has been increasing over the years, largely due to an aging population [1]. In European countries, the prevalence of ILDs varies, with IPF estimates ranging from 1.25 to 23.4 per 100,000 people. A study covering six European countries reported an ILD prevalence between 6.9 and 78 per 100,000 people [1]. The prevalence of IPF in Japan is estimated to be around 10.0 to 27.0 per 100,000 people, with a higher incidence among men and older adults, particularly those aged 75–79 years [2].

Acute exacerbation (AE) is a critical issue for patients with ILD, often leading to a rapid decline in respiratory status and death within a few months. The etiology of acute exacerbations of idiopathic pulmonary fibrosis (IPF) remains an area of active research, and the role of infections in these exacerbations has indeed been the subject of mixed findings [3,4,5,6]. Furthermore, the significance of viral infections in other types of ILDs for triggering AEs remains to be determined.

In the pre-coronavirus disease 2019 (COVID-19) era, our previous study reported that 19.2% of patients experiencing acute exacerbations of ILDs, including IPF, non-IPF, and CTD-associated pneumonia, had viral infections, primarily human herpes virus 7 and cytomegalovirus [7]. However, significant numbers of respiratory viruses were rarely identified in patients with an ILD during that period.

Conversely, during the same timeframe, patients with an exacerbation of asthma (75.3% of inpatients and 19.3% of outpatients) frequently harbored respiratory viruses, such as human rhinovirus (HRV), human metapneumovirus (hMPV), respiratory syncytial virus (RSV), and influenza virus (Inf-V), exhibiting seasonal variations [8]. This highlights disease-specific susceptibility to pathogens.

Following the emergence of severe acute respiratory syndrome coronavirus 2 (SARS-CoV-2), the clinical and radiological similarities between COVID-19-related pneumonia and acute exacerbation (AE) of ILDs prompted the development and widespread adoption of multiplex polymerase chain reaction (PCR) assays for pathogen detection in clinical settings. In this context, our study aimed to adopt a multidisciplinary approach to AEs of ILDs, encompassing the investigation of etiological agents, including SARS-CoV-2, using multiplex PCR, exploring seasonal and regional patterns, and identifying prognostic factors.

## 2. Materials and Methods

### 2.1. Patients and Study Design

We conducted a retrospective cohort study by enrolling hospitalized adult patients experiencing an AE of interstitial pneumonia at Kyorin University Hospital from January 2019 to February 2024. The definition of an AE of interstitial pneumonia (IP) followed the criteria established in a previous report [3]: (1) unexplained onset of dyspnea within 30 days; (2) new bilateral pulmonary ground-glass opacities or consolidations superimposed on a reticular and/or honeycomb pattern on chest computed tomography; (3) acute respiratory symptoms; (4) the absence of pathogenic bacteria in bronchoalveolar lavage fluid; and (5) the exclusion of alternative causes, such as left heart failure and pulmonary embolism. Patients with drug-induced pneumonia or hypersensitivity pneumonia were excluded.

### 2.2. Samples and Clinical Data Collection

Clinical data collected upon admission included age, sex, underlying diseases, vital signs, including delta heart rate (ΔHR)/body temperature (ΔBT) [9], symptoms, use of antifibrotic agents, previous exacerbation of IP, treatment regimens, serum laboratory data, and pulmonary function tests prior to exacerbation, if available. The Δ heart rate and ΔBT were defined as changes in HR and BT between admission and the non-illness state (prior to the AE or post-treatment, if previous data were unavailable). The post-pandemic period was defined as beginning in May 2023, following the lifting of COVID-19-related travel restrictions. Patient characteristics were compared between the pre- and post-COVID-19 pandemic periods.

### 2.3. Definition of Stages

The Ministry of Health, Labour and Welfare of Japan established the criteria for the stages of ILDs [10]. The classification of severity is determined by the following criteria: an arterial blood oxygen pressure (PaO_2_) at rest of 80 Torr or higher is classified as grade I, between 70 Torr and 80 Torr as grade II, between 60 Torr and 70 Torr as grade III, and below 60 Torr as grade IV. For patients with grade I or II PaO_2_ at rest, if the minimum SpO_2_ during a 6 min walk test is less than 90%, the severity is classified as grade III. Additionally, for patients with grade III PaO_2_ at rest, if the minimum SpO_2_ during a 6 min walk test is less than 90%, the severity is classified as grade IV. However, if PaO_2_ at rest is below 70 Torr, the minimum SpO_2_ during the 6 min walk test does not necessarily need to be measured.

**Figure 1:**
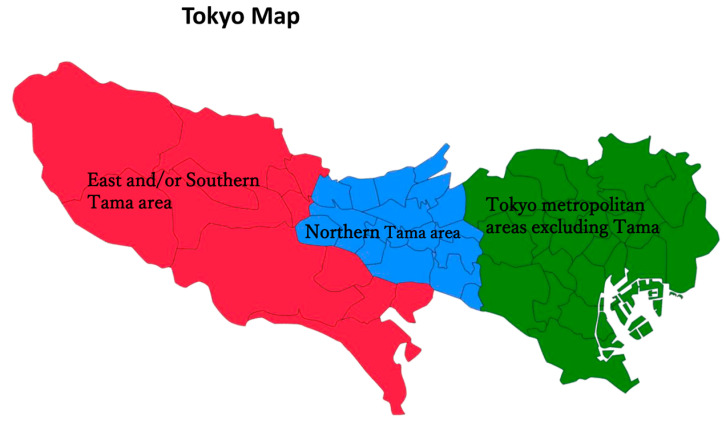
The map of Tokyo consists of three areas: East and Southern Tama area (red color), Northern Tama area (blue color), and Tokyo metropolitan areas (green color).

We analyzed the patients based on seasonal and regional distributions (Northern Tama area, East and/or Southern Tama area, and other Tokyo metropolitan areas, excluding Tama) during the study period (Figure 1). Kyorin University Hospital is in Northern Tama, which is home to approximately 4.23 million residents and covers a vast geographic area.

Nasal swab samples collected upon admission were subjected to multiplex-nested PCR (FilmArray^®^ Respiratory Panel 2.1, bioMérieux Co. Ltd., Tokyo, Japan), which is capable of detecting respiratory viruses such as SARS-CoV-2, adenovirus, human coronaviruses (229E, HKU1, NL63, and OC43), hMPV, HRV/enterovirus, Inf-V A (H1, H1-2009, and H3), Inf-V B, para-Inf-Vs 1-4, and RSV. Bacterial pathogens, including *Bordetella parapertussis*, *Bordetella pertussis*, *Chlamydia pneumoniae*, and *Mycoplasma pneumoniae*, were also included in the panel. The FilmArray^®^ Respiratory Panel 2.1 was introduced in our hospital in December 2020, nine months after the beginning of the COVID-19 pandemic on the Diamond Princess cruise ship in Japan.

This study was approved by the Ethics Committee of Kyorin University (approved number: 2421).

### 2.4. Statistical Analysis

Nonparametric data were analyzed using the Mann-Whitney U test or Wilcoxon signed-rank test. Categorical data were compared using Pearson’s chi-square test. The median overall survival (OS) was estimated using the Kaplan-Meier method with 95% confidence intervals (CIs), and the differences between survival curves were assessed using the log-rank test. Cox proportional hazards regression models were used to calculate hazard ratios (HRs) and 95% CIs. All statistical tests were two-sided, with *p* < 0.05 considered statistically significant. Statistical analyses were performed using SPSS version 25.0 for Windows.

## 3. Results

### 3.1. Patient Characteristics

A total of 93 hospitalized patients with an AE of IP were examined during the study period. The median age was 80 years (interquartile range [IQR]: 74.0–86.0 years), with males comprising 64.5% (*n* = 60), and 66.7% (*n* = 62) were current or former smokers. The percentage of patients in stage I or II was 14.0% (*n* = 13), with a median duration of illness of 4.0 years (IQR: 2.0–5.0 years) (Table 1). Among the idiopathic interstitial pneumonia (IIP) patients, 42 had IPF, 37 had non-IPF (iNSIP), and 14 had connective tissue disease (CTD), including rheumatoid arthritis (*n* = 7), dermatomyositis (*n* = 4), systemic sclerosis (*n* = 2), and systemic lupus erythematosus (*n* = 1). The major comorbidities included cardiac disease (23.7%, *n* = 22), malignant disease (19.3%, *n* = 18), type 2 diabetes mellitus (14.0%, *n* = 13), and chronic obstructive pulmonary disease (14.0%, *n* = 13). Dyspnea was the most common symptom. Upper respiratory tract infections (e.g., nasal discharge or sore throat) were rare, and viral infections were only detected in two patients (SARS-CoV-2), with bacterial infections limited to methicillin-resistant *Staphylococcus aureus* and *Pseudomonas aeruginosa*. The value of ΔHR/ΔBT (median 27.8, IQR: 15.2–56.0) was noted, and most patients had experienced previous episodes of AEs. Before and after the COVID-19 pandemic, the number of patients was 73 and 20, respectively, with a frequency of 1.4 and 2.0 persons per month, suggesting a resurgence of AEs of ILDs.

The characteristics of these patients were comparable, except for the ratios of patients with COPD and NIDDM type 2 (Table 2).

### 3.2. Regional and Seasonal Distribution of Admitted Patients

**Figure 2:**
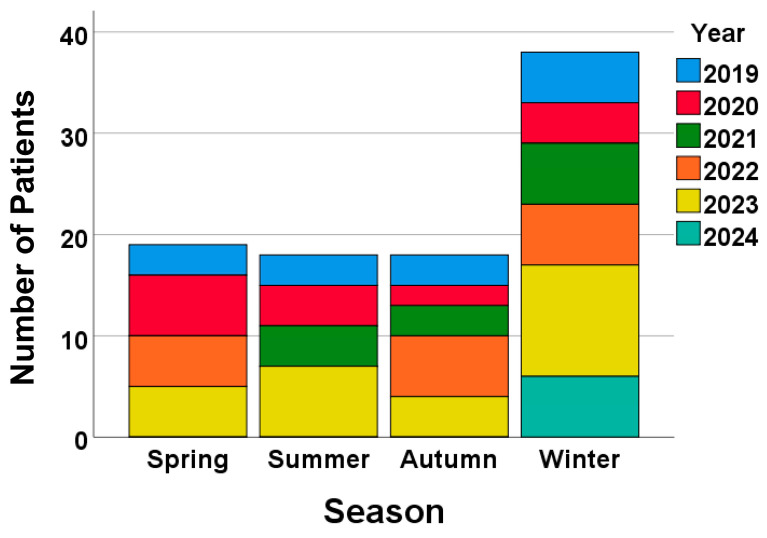
The number of admitted patients increased from the summer of 2023 following the relaxation of COVID-19-related travel restrictions.

The annual and seasonal distribution of admitted patients indicated a resurgence of AEs of ILDs from the summer of 2023 following the relaxation of COVID-19-related travel restrictions in Japan (Figure 2), which allowed people to move across prefectures.

**Figure 3:**
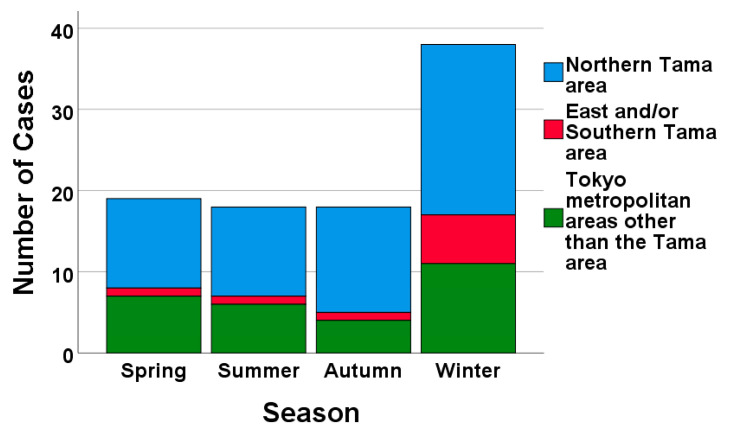
The number of admitted patients was highest in winter, but the proportions of patients in areas remained comparable during the study period.

While the total number of patients was highest in winter, the proportions among the different regions (Northern Tama, East and/or Southern Tama, and other Tokyo metropolitan areas) remained consistent across regions throughout the study period (Figure 3).

### 3.3. Various Parameters on Admission between Survivor and Deceased Groups

The survivor group (N = 48) and the deceased group (N = 45) were similar in terms of age, sex, duration of illness, body mass index, smoking status, and the prevalence of underlying diseases or comorbidities (Table 3). However, the proportion of tachypnea (respiratory rate [RR] ≥ 30 breaths/min) was significantly higher in the deceased group than in the survivor group (33.3% vs. 12.5%, *p* = 0.025). On admission, the deceased group also exhibited significantly higher white blood cell counts (median 11,000/µL, IQR: 9100–13,650/µL, *p* = 0.004), lactate dehydrogenase (LDH) levels (median 384 IU/L, IQR: 295–517, *p* = 0.004), and surfactant protein D (SP-D) levels (median 401 IU/L, IQR: 270–719, *p* = 0.007) compared to the survivor group (WBC: median 9400/µL, IQR: 7275–11,925/µL; LDH: median 317 IU/L, IQR: 251–363; SP-D: median 252 IU/L, IQR: 127–434). Serum LDH levels ≥ 350 IU/L and SP-D levels ≥ 314 IU/L were more frequent in deceased patients than survivors (LDH: 51.1% vs. 39.6%, *p* = 0.005; SP-D ≥ 314 IU/L: 40.4% vs. 29.2%, *p* = 0.046). Although pulmonary function tests were not performed for all patients (survivor: *n* = 30, 62.5%; deceased: *n* = 15, 33.3%), the results were comparable between the groups.

### 3.4. Comparison of 30-Day Survival Probabilities on a Kaplan–Meier Plot Based on the Presence of Tachypnea (RR ≥ 30 Breaths/Min) and Elevated LDH Levels (≥350 IU/L)

**Figure 4:**
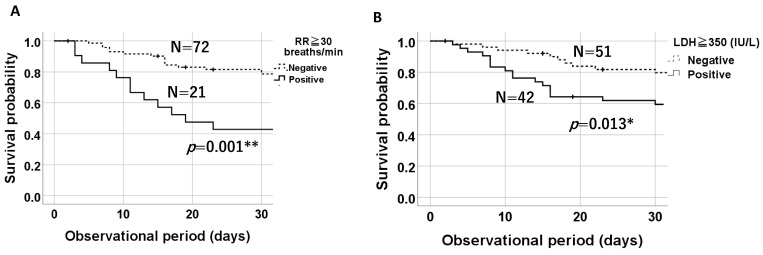
Kaplan–Meier plots demonstrating that tachypnea (RR ≥ 30 breaths/min) (**A**) and serum LDH levels ≥ 350 IU/L (**B**) were significant prognostic factors for 30-day survival probabilities. * means *p* < 0.05, ** means *p* < 0.01.

Kaplan–Meier survival curves over 30 days showed that patients with tachypnea (RR ≥ 30 breaths/min) had a 30-day survival rate of 42.9%, compared to 76.9% in those without tachypnea (*p* = 0.001) (Figure 4A). Similarly, patients with an LDH level ≥ 350 IU/L on admission exhibited significantly lower survival probabilities than those with an LDH level < 350 IU/L (positive: *n* = 42, 54.8%; negative: *n* = 51, 80.4%, *p* = 0.013) based on the log-rank test (Figure 4B).

### 3.5. Prognostic Factors for an AE of IP

The multivariate analysis adjusted for age and sex demonstrated that serum LDH levels ≥ 350 IU/L and tachypnea (RR ≥ 30 breaths/min) were identified as risk factors for 30-day mortality, with hazard ratios of 4.0 (95% CI: 1.45–11.0, *p* = 0.007) and 4.85 (95% CI: 1.61–14.61, *p* = 0.005), respectively (Table 4). The Cox regression analysis further confirmed serum LDH levels ≥350 IU/L and tachypnea (RR ≥ 30 breaths/min) as prognostic factors for mortality, with hazard ratios of 2.78 (95% CI: 1.48–5.24, *p* = 0.001) and 3.33 (95% CI: 1.71–6.49, *p* < 0.001), respectively (Table 5), but not serum SP-D levels ≥ 314 IU/L (HR 2.009, 95% CI: 0.90–4.51, *p* = 0.09).

## 4. Discussion

This study demonstrated that AEs of ILDs predominantly occurred in winter, particularly increasing from the summer of 2023, shortly after the lifting of COVID-19-related travel restrictions in Japan. Infections were infrequently detected during the study. Serum LDH levels ≥ 350 IU/L and tachypnea (RR ≥ 30) on admission emerged as potent prognostic factors for mortality, indicating the need for more intensive treatment (e.g., steroid pulse therapy and/or other immunosuppressive drugs).

Regarding infectious agents, Mostafaei et al. [11] reported a pooled prevalence of viral and bacterial infections in patients with IPF of 53.72% and 31.21%, respectively, based on a random-effects meta-analysis. However, these infections may not always act as direct risk factors for AEs of IPF but may be involved in the pathogenesis of interstitial pneumonia. Pathogen detection rates varied due to geographical differences, techniques for viral/bacterial detection, and the types of biological samples used. Our study did not perform PCR testing for some viruses, such as cytomegalovirus (CMV), Epstein–Barr virus (EBV), and human herpesvirus (HHV) 7 and 8, as in our previous study [8] or other studies [12], potentially affecting the detection rate. Nonetheless, the low frequency of upper respiratory tract infection symptoms and the absence of viruses other than SARS-CoV-2 raise the hypothesis that the higher detection rates reported in comprehensive studies may correspond to the reactivation of certain viruses (e.g., HHV7, HHV8, CMV, and EBV) in immunocompromised hosts or disease progression of ILDs, although their roles in AEs remain uncertain.

In terms of the annual and seasonal distributions of AEs of ILDs, the total number of cases was higher in winter than in other seasons and appeared to increase from summer 2023 following the easing of COVID-19-related travel restrictions. Previous studies focusing on AEs of IPF have shown higher exacerbation rates in winter to spring [13,14] or specifically in winter [15]. Although our study included patients with and without IPF (e.g., idiopathic NSIP and CTD-ILD), winter was the predominant season for AEs of ILDs. The reason for this seasonal predilection remain unclear; however, unidentified pathogens may contribute to the occurrence of AEs. Regarding regional distributions, we anticipated observing regional variations in patient referrals to our hospital, given that it serves as the referral center for Mitaka City. However, the regional variations appeared unchanged before and after the COVID-19 pandemic. Based on these data alone, we cannot assess the effects on healthcare delivery.

For clinicians, vital signs play a crucial role in hypothesizing etiologies. In healthy individuals, the normal physiological response involves an increase in heart rate by approximately 10 beats/min per 0.55 °C rise in body temperature, resulting in an expected ΔHR/ΔBT ≤ 20. However, the AE of ILDs in our study exhibited higher ΔHR/ΔBT values without bacterial infection, suggesting that this rule may not apply to AE of ILDs with bacterial infections. Conversely, RR ≥ 30 breaths/min on admission could serve as a pivotal prognostic sign for short-term (30-day) or long-term mortality, along with an elevated serum LDH level (≥350 IU/L). To the best of our knowledge, the severity of tachypnea in AEs of ILDs has rarely been reported; however, its correlation with mortality rates resembles that of community-acquired pneumonia [16].

The serum KL-6 level has been identified as a valuable biomarker for both diagnostic purposes and assessing the severity of ILDs in children with CTD [17], as well as in adults with CTD [18]. A systematic review of adults with CTD (rheumatoid arthritis-associated ILD) indicated that KL-6, SP-D, and interleukin-6 are associated with all-cause mortality [19]. In clinically amyopathic adults with dermatomyositis, Gono et al. reported that ferritin predicts the disease severity and prognosis of amyopathic dermatomyositis [20]. Furthermore, Zou J et al. reported that elevated serum ferritin levels and the extent of lung involvement, as calculated by high-resolution CT, were independent significant factors for 1-year mortality [21]. However, this was not the case for LDH levels, based on a multivariable analysis using Cox proportional hazards regression models.

On the other hand, Kishaba et al. found that high LDH (>280 IU/L) and KL-6 levels (>1000 IU/L) predict the 3-month mortality rate due to AEs of IPF [22]. Murohashi et al. reported the utility of the Charlson Comorbidity Index, sex, and serum LDH levels as mortality prediction tools for acute or subacute idiopathic interstitial pneumonia and AEs of CVD-IP [23].

The annual incidence of AEs of IPF is reported to be 5–19%, and AEs are of milder forms of IPF are less common. However, the post-exacerbation mortality of ILDs is reported to range from 33–83% with hospital mortality rates of 50–100% for patients with CTD-ILDs and 75–100% for patients with hypersensitivity pneumonitis [24]. Furthermore, many cases of ILDs rarely showed external triggers, including infection. Therefore, the early recognition of AE-ILDs, followed by urgent treatment using simple clinical parameters, is essential.

This study had limitations: (1) It was conducted as an observational study at a single center. (2) The FilmArray^®^ Respiratory Panel 2.1 was applied to only 79 admitted patients from January 2020 to 2023, thus not covering all patients in the study. In addition, some viruses detected in previous studies were not examined. (3) The relatively small number of enrolled patients was due to the low incidence of AEs of ILDs. Nevertheless, this study provides clinical evidence that both serum LDH levels (≥350 IU/L) and tachypnea (RR ≥ 30 breaths/min) can serve as simple yet potent prognostic factors and that AEs of ILDs exhibit a paucity of infections with a seasonal predilection for winter.

## 5. Conclusions

Acute exacerbations of ILDs predominantly occur in winter, with limited evidence of bacterial and viral infections, even during the COVID-19 era. A serum LDH level ≥ 350 IU/L and tachypnea (RR ≥ 30 breaths/min) represent simple yet crucial prognostic factors for mortality.

## Figures and Tables

**Table 1:** Patient characteristics.

Number of Patients (*n* = 93)	
Age	80.0 (74.0–86.0)
Sex (Male)	64.5% (*n* = 60)
Smoking	
Ex or Current	66.7% (*n* = 62)
IIPs	
IPF	44.1% (*n* = 42)
non-IPF (iNSIP)	40.9% (*n* = 37)
CTD-ILD (N = 14)	
RA	7.5% (*n* = 7)
DM	4.3% (*n* = 4)
SLE	1.1% (*n* = 1)
SSc	2.2% (*n* = 2)
Stage I or II	14.0% (*n* = 13)
Duration of illness (years)	4.0 (2.0–5.0)
Comorbidities	
Asthma	0% (*n* = 0)
COPD	14.0% (*n* = 13)
Cardiac diseases	23.7% (*n* = 22)
NIDDM type 2	14.0% (*n* = 13)
Maintenance hemodialysis	1.1% (*n* = 1)
Malignant diseases	19.3% (*n* = 18)
Respiratory viruses *	
SARS-CoV-2	2.5% (*n* = 2)
Bacteria	
MRSA	1.1% (*n* = 1)
*Pseudomonas aeruginosa*	1.1% (*n* = 1)
Vital signs	
BT (°C)	37.0 ± 0.76
RR (breaths/min)	24.0 (18.0–28.0)
ΔHR/ΔBT	27.8 (15.2–56.0)
SpO2 (%)	82.1 (78.6–85.3)
BMI	21.5 (18.7–24.1)
Symptoms	
nasal discharge	5.4% (*n* = 5)
sore throat	1.1% (*n* = 1)
dyspnea	73.1% (*n* = 68)
Antifibrotic agents	9.7% (*n* = 9)
Previous episodes of AEs	14.0% (*n* = 13)

* The FilmArray^®^ Respiratory Panel 2.1 was applied for 79 admitted patients from Jan 2020 to 2023. Data are expressed as percentages (numbers) or medians (interquartile ranges). The stage classification was based on the Japanese Respiratory Society guideline 2022. AE, acute exacerbation; BMI, body mass index; BT, body temperature; COPD, chronic obstructive pulmonary disease; CTD-ILD, connective tissue disease–interstitial lung disease; IIPs, idiopathic interstitial pneumonias; IPF, idiopathic pulmonary fibrosis; non-IPF (iNSIP), idiopathic nonspecific interstitial pneumonia; MRSA, methicillin-resistant *Staphylococcus aureus*; NIDDM, non-insulin-dependent diabetes mellitus; RA, rheumatoid arthritis; RR, respiratory rate; SARS-CoV-2, severe acute respiratory syndrome coronavirus 2; SLE, systemic lupus erythematosus; SSc, systemic scleroderma.

**Table 2:** Patient characteristics before and after the pandemic.

	Before the Pandemic (*n* = 73)	After the Pandemic (*n* = 20)	*p* Value
Age	80 (70–83)	79 (70–87)	0.918
Sex (Male)	68.5% (*n* = 50)	50.0% (*n* = 10)	0.186
Smoking			
Ex or Current	72.6% (*n* = 53)	50.0%(*n* = 10)	0.065
IIPs	84.9% (*n* = 62)	85.0% (*n* = 17)	1
IPF	46.6% (*n* = 34)	35.0% (*n* = 7)	0.411
non-IPF (iNSIP)	38.4% (*n* = 28)	50.0%(*n* = 10)	0.414
CTD-ILD	15.1% (*n* = 11)	15.0% (*n* = 3)	1
RA	8.2% (*n* = 6)	5.0% (*n* = 1)	1
DM PM	6.8% (*n* = 5)	0% (*n* = 0)	0.581
SLE	1.4% (*n* = 1)	0% (*n* = 0)	1
SSc	0% (*n* = 0)	10.0% (*n* = 2)	0.044
Stage I or II	17.8% (*n* = 13)	0% (*n* = 0)	0.052
Duration of illness (years)	4.0 (3.0–5.0)	4.0 (1.0–6.0)	0.501
Comorbidities			
Asthma	0% (*n* = 0)	0% (*n* = 0)	N.D
COPD	8.2%(*n* = 6)	35.0%(*n* = 7)	0.006
Cardiac diseases	19.2% (*n* = 14)	40.0% (*n* = 8)	0.074
NIDDM type 2	8.2% (*n* = 6)	35.0% (*n* = 7)	0.006
Maintenance hemodialysis	1.4% (*n* = 1)	0% (*n* = 0)	1
Malignant diseases	17.8% (*n* = 13)	25.0% (*n* = 5)	0.526
Respiratory viruses			
SARS-CoV-2	0% (*n* = 0)	10.0% (*n* = 2)	0.044
Bacteria	1.4% (*n* = 1)	5.0% (*n* = 1)	0.386
MRSA	1.4% (*n* = 1)	0% (*n* = 0)	1
Pseudomonas aeruginosa	0% (*n* = 0)	5.0% (*n* = 1)	0.215
Vital signs			
BT (°C)	36.6 (36.6–37.2)	36.7 (36.6–37.1)	0.191
RR (breaths/min)	22 (18–28)	24 (20–32)	0.209
ΔHR/ΔBT	36.3 (15.9–190.3)	16.5 (7.5–32.0)	0.109
SpO2 (%)	88.0 (82.0–95.0)	86.0 (81.0–91.0)	0.784
BMI	22.4 (19.4–25.3)	21.5 (16.0–26.0)	0.726
Symptoms			
nasal discharge	5.5% (*n* = 4)	5.0% (*n* = 1)	1
sore throat	1.4% (*n* = 1)	0% (*n* = 0)	1
dyspnea	1.4% (*n* = 1)	0% (*n* = 0)	0.085
Antifibrotic agents	12.3% (*n* = 9)	0% (*n* = 0)	0.197
Previous episodes of AEs	16.4% (*n* = 12)	5.0% (*n* = 1)	0.285

The FilmArray^®^ Respiratory Panel 2.1 was applied for 79 admitted patients from Jan 2020 to 2023. Data are expressed as percentages (numbers) or medians (interquartile ranges). The stage classification was based on the criteria of idiopathic interstitial pneumonia according to the Ministry of Health, Labour and Welfare of Japan [10]. AE, acute exacerbation; BMI, body mass index; BT, body temperature; COPD, chronic obstructive pulmonary disease; CTD-ILD, connective tissue disease–interstitial lung disease; IIPs, idiopathic interstitial pneumonias; IPF, idiopathic pulmonary fibrosis; non-IPF (iNSIP), idiopathic nonspecific interstitial pneumonia; MRSA, methicillin-resistant *Staphylococcus aureus*; NIDDM, non-insulin-dependent diabetes mellitus; RA, rheumatoid arthritis; RR, respiratory rate; SARS-CoV-2, severe acute respiratory syndrome coronavirus 2; SLE, systemic lupus erythematosus; SSc, systemic scleroderma.

**Table 3:** Comparison between survivor and deceased groups.

All Patients (*n* = 93)	Survivors (*n* = 48)	Deceased (*n* = 45)	*p* Value
Age	79 (70–84)	81 (72–85)	0.142
Sex	62.5% (*n* = 30)	66.7% (*n* = 30)	0.829
Duration of illness (years)	4.0 (2.8–5.3)	4.0 (2.5–5.0)	0.634
BMI	21.6 (18.7–24.6)	21.5 (19.0–24.0)	0.860
Smoker (ex or current)	66.7% (*n* = 32)	68.9% (*n* = 31)	0.829
SpO2 (%)	90.0 (83.0–94.0)	84.0 (73–91.0)	0.008 **
Hypoxemia (<SpO2 94%)	79.2% (*n* = 48)	88.9% (*n* = 45)	0.264
RR (breaths/min)	20 (18–22)	30 (24–36)	0.013 *
HR (beats/min)	91(80–106)	85 (78–102)	0.997
RR ≥ 20 (breaths/min)	58.3% (*n* = 28)	73.3% (*n* = 33)	0.190
RR ≥ 30 (breaths/min)	12.5%(*n* = 6)	33.3% (*n* = 15)	0.025 *
ΔHR/ΔBT	27.8 (19.5–117.5)	35.0(9.6–188)	0.694
Body temperature (°C)	36.7 (36.6–37.1)	36.6 (36.6–36.7)	0.375
IIPs	85.4% (*n* = 41)	84.4% (*n* = 38)	1.0
IPF	43.8% (*n* = 21)	44.4% (*n* = 20)	0.824
non-IPF (iNSIP)	41.7% (*n* = 20)	40.0% (*n* = 18)	1.0
CTD-ILD			
RA	6.3% (*n* = 3)	11.1% (*n* = 5)	0.205
DM	2.1% (*n* = 1)	0% (*n* = 0)	0.330
SLE	2.1% (*n* = 1)	0% (*n* = 0)	0.330
Stage I or II *	10.4% (*n* = 5)	17.8% (*n* = 8)	0.755
Comorbidities			
COPD	12.5% (*n* = 6)	15.6% (*n* = 7)	0.769
Cardiac diseases	16.7% (*n* = 8)	31.1% (*n* = 14)	0.149
NIDDM type 2	16.7% (*n* = 8)	11.1% (*n* = 5)	0.440
Maintenance hemodialysis	2.1% (*n* = 1)	0% (*n* = 0)	0.330
Malignant diseases	14.5% (*n* = 7)	22.9% (*n* = 11)	0.871
Previous episodes of AE	12.5% (*n* = 6)	15.6% (*n* = 7)	0.769
HOT on admission	22.9% (*n* = 11)	17.8% (*n* = 8)	0.612
Hospital days	28.0 (21.0–39.0)	18.0 (13.0–33.0)	0.003 **
Duration of hypoxemia or needs more O2 supply than usual (days)	9.5 (3.0–13.0)	10.0(5.0–16.0)	0.106
Respiratory viruses	4.2% (*n* = 2)	0% (*n* = 0)	0.495
Bacteria	0% (*n* = 0)	4.4% (*n* = 2)	0.231
Symptoms			
Nasal discharge	6.3% (*n* = 3)	4.4% (*n* = 2)	1
Sore throat	2.1% (*n* = 1)	0% (*n* = 0)	1
Dyspnea on efforts	68.8% (*n* = 33)	77.8% (*n* = 35)	0.358
Antifibrotic agents	8.3% (*n* = 4)	11.1% (*n* = 5)	0.735
Treatments			
IVCY	18.8% (*n* = 9)	42.2% (*n* = 19)	0.023 *
mPSL pulse	81.3% (*n* = 39)	91.1% (*n* = 41)	0.235
Laboratory data			
WBC	9400 (7275–10925)	11,000 (9100–13,650)	0.004 **
Monocyte	7.1 (6.1–10.9)	6.2 (5.0–7.5)	0.010 *
Albumin	3.1 (2.7–3.4)	2.9 (2.5–3.2)	0.101
Plt	24.9 (18.1–32.2)	20.4 (15.2–30.2)	0.089
CRP	7.51 (3.60–12.8)	10.2(5.5–14.4)	0.114
LDH	317 (251–363)	384 (295–517)	0.004 **
LDH ≥ 350	39.6% (*n =* 19)	51.1% (*n* = 23)	0.005 **
KL-6	989 (691–1707)	988 (547–2145)	0.913
SP-D	252 (127–434)	401 (270–719)	0.007 **
SP-D ≥ 314	29.2% (*n* = 14)	40.4% (*n* = 18)	0.046 *
Pulmonary function test			
VC	70.8 (55.2–86.6)	82.6 (60.9–90.2)	0.639
FVC	72.5 (56.4–92.6)	80.9 (63.1–84.7)	0.736
FEV1.0%	84.2 (80.1–86.9)	79.9 (73.1–86.3)	0.691
%FEV1.0	74.8 (59.9–92.8)	74.2 (70.0–87.0)	0.791
%DLCO	41.6 (33.3–54.3)	57.4 (30.0–70.6)	0.336
%DLCO/VA	50.0(40.0–75.3)	59.4 (49.8–74.3)	0.428

AE, acute exacerbation; BMI, body mass index; BT, body temperature; CTD-ILD, connective tissue disease related to interstitial lung disease; COPD, chronic obstructive lung disease; DM, dermatomyositis; DLCO, diffusing capacity of the lungs for carbon monoxide; DLCO/VA, diffusing capacity of the lungs for carbon monoxide/alveolar volume; FEV1.0%, forced expiratory volume in one second/forced vital capacity; %FEV1.0, percent predicted forced expiratory volume in one second; FVC, forced vital capacity; IIPs, idiopathic interstitial pneumonia; IPF, idiopathic pulmonary fibrosis; mPSL, methylprednisolone; NIDDM, non-insulin dependent diabetes mellitus; RA, rheumatoid arthritis; RR, respiratory rate; SLE, systemic lupus erythematosus; SpO2, oxygen saturation; IVCY, intravenous cyclophosphamide; VC, vital capacity, Pulmonary function tests were available for 62.5% of the survivor group (*n* = 30) and 33.3% of the deceased group (*n* = 15). * means *p* < 0.05, ** means *p* < 0.01.

**Table 4:** Multivariate analysis of 30-day mortality.

Parameter	Hazard Ratio (95%CI)	*p* Value
Age, yr	1.036 (0.974–1.103)	0.262
Male sex	1.538 (0.493–4.801)	0.459
LDH ≥ 350 (IU/L)	3.997(1.452–11.0)	0.007 **
RR ≥ 30 (breaths/min)	4.854 (1.613–14.609)	0.005 **

** means *p* < 0.01.

**Table 5:** Cox regression analysis of mortality.

Parameter	Hazard Ratio (95%CI)	*p* Value
Age, yr	1.036 (0.997–1.076)	0.069
Male sex	2.022 (0.961–4.254)	0.064
LDH ≥ 350 (IU/L)	2.783 (1.480–5.235)	0.001 **
RR ≥ 30 (breaths/min)	3.332 (1.710–6.492)	<0.001 ***

** means *p* < 0.01, *** means *p* < 0.001.

## Data Availability

The data used and/or analyzed in this study are available from the corresponding author on reasonable request. All data generated or analyzed during this study are included in this manuscript, and the database is available from the corresponding author (saraya@ks.kyorin-u.ac.jp) upon reasonable request.

## References

[1] Jeganathan N., Sathananthan M. (2022). The prevalence and burden of interstitial lung diseases in the USA. ERJ Open Res..

[2] Kondoh Y., Suda T., Hongo Y., Yoshida M., Hiroi S., Iwasaki K., Takeshima T., Homma S. (2022). Prevalence of idiopathic pulmonary fibrosis in Japan based on a claims database analysis. Respir. Res..

[3] Collard H.R., Moore B.B., Flaherty K.R., Brown K.K., Kaner R.J., King T.E., Lasky J.A., Loyd J.E., Noth I., Olman M.A. (2007). Acute exacerbations of idiopathic pulmonary fibrosis. Am. J. Respir. Crit. Care Med..

[4] Tang Y.W., Johnson J.E., Browning P.J., Cruz-Gervis R.A., Davis A., Graham B.S., Brigham K.L., Oates J.A., Loyd J.E., Stecenko A.A. (2003). Herpesvirus DNA is consistently detected in lungs of patients with idiopathic pulmonary fibrosis. J. Clin. Microbiol..

[5] Ushiki A., Yamazaki Y., Hama M., Yasuo M., Hanaoka M., Kubo K. (2014). Viral infections in patients with an acute exacerbation of idiopathic interstitial pneumonia. Respir. Investig..

[6] Wootton S.C., Kim D.S., Kondoh Y., Chen E., Lee J.S., Song J.W., Huh J.W., Taniguchi H., Chiu C., Boushey H. (2011). Viral infection in acute exacerbation of idiopathic pulmonary fibrosis. Am. J. Respir. Crit. Care. Med..

[7] Saraya T., Kimura H., Kurai D., Tamura M., Ogawa Y., Mikura S., Sada M., Oda M., Watanabe T., Ohkuma K. (2018). Clinical significance of respiratory virus detection in patients with acute exacerbation of interstitial lung diseases. Respir. Med..

[8] Saraya T., Kimura H., Kurai D., Ishii H., Takizawa H. (2017). The molecular epidemiology of respiratory viruses associated with asthma attacks. A single-center observational study in Japan. Medicine.

[9] Hamano J., Tokuda Y. (2017). Changes in vital signs as predictors of bacterial infection in home care: A multi-center prospective cohort study. Postgrad. Med..

[10] The Ministry of Health LaWoJ Idiopathic Interstitial Pneumonia; 2024. https://www.nanbyou.or.jp/entry/302.

[11] Mostafaei S., Sayad B., Azar M.E.F., Doroudian M., Hadifar S., Behrouzi A., Riahi P., Hussen B.M., Bayat B., Nahand J.S. (2021). The role of viral and bacterial infections in the pathogenesis of IPF: A systematic review and meta-analysis. Respir. Res..

[12] Sheng G., Chen P., Wei Y., Yue H., Chu J., Zhao J., Wang Y., Zhang W., Zhang H.L. (2020). Viral Infection Increases the Risk of Idiopathic Pulmonary Fibrosis: A Meta-Analysis. Chest.

[13] Collard H.R., Yow E., Richeldi L., Anstrom K.J., Glazer C. (2013). Suspected acute exacerbation of idiopathic pulmonary fibrosis as an outcome measure in clinical trials. Respir. Res..

[14] Simon-Blancal V., Freynet O., Nunes H., Bouvry D., Naggara N., Brillet P.Y., Denis D., Cohen Y., Vincent F., Valeyre D. (2012). Acute exacerbation of idiopathic pulmonary fibrosis: Outcome and prognostic factors. Respiration.

[15] Yamazoe M., Tomioka H. (2018). Acute exacerbation of idiopathic pulmonary fibrosis: A 10-year single-centre retrospective study. BMJ Open Respir. Res..

[16] Shindo Y., Ito R., Kobayashi D., Ando M., Ichikawa M., Goto Y., Fukui Y., Iwaki M., Okumura J., Yamaguchi I. (2015). Risk factors for 30-day mortality in patients with pneumonia who receive appropriate initial antibiotics: An observational cohort study. Lancet Infect. Dis..

[17] El-Beheidy R., Domouky A.M., Zidan H., Amer Y.A. (2021). Serum KL-6 as predictive and prognostic marker of interstitial lung disease in childhood connective tissue diseases: A pilot study. Reumatismo.

[18] Zheng P., Zheng X., Takehiro H., Cheng Z.J., Wang J., Xue M., Lin Q., Huang Z., Huang H., Liao C. (2021). The Prognostic Value of Krebs von den Lungen-6 and Surfactant Protein-A Levels in the Patients with Interstitial Lung Disease. J. Transl. Int. Med..

[19] Groseanu L., Nita C. (2024). A Systematic Review of the Key Predictors of Progression and Mortality of Rheumatoid Arthritis-Associated Interstitial Lung Disease. Diagnostics.

[20] Gono T., Kawaguchi Y., Satoh T., Kuwana M., Katsumata Y., Takagi K., Masuda I., Tochimoto A., Baba S., Okamoto Y. (2010). Clinical manifestation and prognostic factor in anti-melanoma differentiation-associated gene 5 antibody-associated interstitial lung disease as a complication of dermatomyositis. Rheumatology.

[21] Zou J., Guo Q., Chi J., Wu H., Bao C. (2015). HRCT score and serum ferritin level are factors associated to the 1-year mortality of acute interstitial lung disease in clinically amyopathic dermatomyositis patients. Clin. Rheumatol..

[22] Kishaba T., Tamaki H., Shimaoka Y., Fukuyama H., Yamashiro S. (2014). Staging of acute exacerbation in patients with idiopathic pulmonary fibrosis. Lung.

[23] Murohashi K., Hara Y., Saigusa Y., Kobayashi N., Sato T., Yamamoto M., Kudo M., Kaneko T. (2019). Clinical significance of Charlson comorbidity index as a prognostic parameter for patients with acute or subacute idiopathic interstitial pneumonias and acute exacerbation of collagen vascular diseases-related interstitial pneumonia. J. Thorac. Dis..

[24] Kolb M., Bondue B., Pesci A., Miyazaki Y., Song J.W., Bhatt N.Y., Huggins J.T., Oldham J.M., Padilla M.L., Roman J. (2018). Acute exacerbations of progressive-fibrosing interstitial lung diseases. Eur. Respir. Rev..

